# Market frictions: A unified model of search costs and switching costs

**DOI:** 10.1016/j.euroecorev.2012.05.007

**Published:** 2012-08

**Authors:** Chris M. Wilson

**Affiliations:** School of Business and Economics, Loughborough University, United Kingdom

**Keywords:** Search costs, Switching costs, Consumer search, Oligopoly, Pricing

## Abstract

It is well known that search costs and switching costs can create market power by constraining the ability of consumers to change suppliers. While previous research has examined each cost in isolation, this paper demonstrates the benefits of examining the two types of friction in unison. The paper shows how subtle distinctions between the two costs can provide important differences in their effects upon consumer behaviour, competition and welfare. In addition, the paper also illustrates a simple empirical methodology for estimating separate measures of both costs, while demonstrating a potential bias that can arise if only one cost is considered.

## Highlights

► We show the benefits of studying search costs and switching costs in unison. ► The two costs have different effects on competition and welfare. ► In most cases, search costs are the more anti-competitive and welfare damaging. ► We also offer a methodology to estimate separate measures of the two costs.

## 1 Introduction

As recognised by the 2010 Nobel prize, the study of market frictions has generated rich insights in many areas of economics, especially macroeconomics, labour economics and monetary theory.^1^ In industrial organisation, analysis has focussed on understanding how market frictions can create a source of market power by restricting consumers' ability to change suppliers. Two different forms of friction have been studied. One literature has considered the search costs that consumers face in gathering information about alternative suppliers, while another literature has focussed on the switching costs that consumers may incur as a direct result of changing suppliers. Examples of the latter include additional effort, service disruption, reduced compatibility and lost loyalty discounts.^2^

Surprisingly, these two literatures have remained largely independent of each other. As a consequence, very little is known about the potential differences or interactions between the two forms of friction. This is an important omission because in many markets, consumers are often constrained by *both* search costs and switching costs. For example, in order to change suppliers in a market for a financial product or utility, consumers may have to first spend time searching for information about an alternative, before then incurring switching costs by completing the necessary paperwork. Indeed, the importance of considering both forms of friction within the same market is highlighted by a number of competition policy investigations, not least in the banking sector, where the UK and EU authorities remain concerned over both the transparency of price information and difficulties within the switching process (OFT, 2009; EC, 2009).^3^

In contrast to the existing literature, this paper demonstrates the benefits of examining the effects of search costs and switching costs in unison. It makes two contributions. The main contribution consists of the construction of an oligopoly model where consumers can be constrained by both forms of friction. The model shows how subtle distinctions between the two costs can provide important differences in their effects on consumer behaviour, competition and welfare. Indeed, in most cases, the levels of competition and welfare are shown to be more sensitive to the level of search costs than the level of switching costs. In relation to the concerns over European bank markets or indeed any other market, this finding suggests that a policy intervention to reduce search costs may provide larger benefits to competition and welfare than an equivalent reduction in switching costs. Subject to their relative resource costs, government authorities may prefer policies to improve consumers' information rather than to ease the switching process.

As a secondary contribution, the paper then uses some results from the theoretical model to offer a simple empirical methodology for simultaneously estimating measures of both costs. In contrast to most of the previous literature which focuses on estimating only one of the costs (as reviewed in the next section), our methodology emphasises the potential importance of accounting for both frictions. Indeed, by attributing any market imperfection to only one cost, we demonstrate how some ‘single-cost’ measures can exhibit an upward bias.

The theoretical model is presented in Sections 4 and 5. It introduces search costs and switching costs into a version of Perloff and Salop's (1985) model of oligopoly with differentiated products. The market is assumed to be mature in the sense that each consumer already faces costs of searching and switching away from their given supplier. In particular, at a cost of *c* per firm, consumers can search firms sequentially in order to learn each firm's price and their willingness to pay for each firm's product. After having stopped searching, each consumer must then decide whether to remain with their existing supplier or to trade with a previously searched alternative firm for an extra switching cost, *s*. We present the equilibrium of a game where firms select prices and consumers simultaneously select their search and switching strategies. The equilibrium generalises the results of some standard models that consider only one cost, such as Wolinsky (1986) and Anderson and Renault (1999).^4^

Section 6 then examines some comparative statics. We first compare how the two costs affect the equilibrium price. While a unit increase in either cost raises the equilibrium price, the paper shows that the underlying mechanisms differ substantially. A rise in search costs deters consumers from starting to search beyond their existing firm and prompts searching consumers to search fewer firms. A rise in switching costs also discourages consumers from initiating any search activity, but it has no effect on the number of firms that searching consumers choose to search. Instead, a rise in switching costs deters consumers that have searched the entire market from switching to an alternative firm. The paper demonstrates that these mechanisms are so different that in most cases, search costs have the consistently more powerful effect on market power. We then consider how the two costs affect welfare. This is more complex as changes in either cost affect not only the equilibrium price but also consumers' searching, switching and purchase decisions. However, the paper shows that whenever search costs have the relatively larger effect on the equilibrium price, they also have the relatively larger impact on consumer surplus, profits and total welfare.

In Section 7, we investigate the robustness of the results in a number of regards. The most important of these concerns the introduction of dynamic competition. While it is customary to think of switching costs in a dynamic setting, the main model is static and focuses on a mature market. However, we argue that any dynamic effects are only likely to strengthen our findings due to the following reasoning. Switching costs are, by definition, only active after a consumer has made an initial market purchase. Consequently, it is well known that the introduction of dynamic competition often erodes the anti-competitive effects of switching costs by inducing firms to compete fiercely for the future profits of new consumers that are yet to be locked-in (Farrell and Klemperer, 2007). However, as later discussed, search costs are often active both before and after an initial market purchase and so the anti-competitive effects of search costs are not eroded in the same way and search costs remain the more welfare damaging.

Finally, Section 8 uses the model to present a quick methodology for simultaneously calculating some ‘back of the envelope’ measures of both search and switching costs. This methodology builds on a useful feature of the theoretical model where consumers differ in their expost equilibrium behaviour - a fraction of consumers do not search, a fraction of consumers search and switch and a fraction of consumers search but refrain from switching. Using data from eight UK markets, we then show how one can use some of these equilibrium restrictions with aggregate consumer survey data to recover separate measures for the two costs.

## 2 Previous literature

Two previous papers have offered a theoretical analysis of search costs and switching costs. Schlesinger and von der Schulenberg (1991) analyse a circular city model where a number of entrants are located evenly between some market incumbents. Consumers must incur a search cost to discover an entrant's price and then further incur a switching cost to trade with the entrant. Their paper is substantially more restrictive than ours and fails to capture the full effects of the two costs in two respects. First, the circular model implies that consumers only consider purchasing from the entrant or the incumbent adjacent to their location and thus rules out the possibility of incurring search costs across multiple firms. Further, the model produces the unrealistic outcome that all consumers who search in equilibrium also decide to switch, such that consumers artificially view the two costs as equivalent.^5^ Sturluson (2002) considers competition between an entrant and an incumbent. Increases in search costs or switching costs are shown to raise prices but no direct comparison of their relative effects is provided. Any potential results are also limited by the focus on a duopoly which minimises the role of multiple searches, and by the fact that equilibrium existence requires a tight parameter condition. In contrast to these papers, the richness of our model allows a detailed comparison of all the effects of the two frictions, and not just on market competition, but also on welfare.

The empirical literature has received somewhat more previous attention. In addition to some studies that use reduced form analysis to analyse both costs,^6^ several papers perform structural estimations of the actual value of either search costs (e.g. Hong and Shum, 2006; Moraga-González and Wildenbeest, 2008; Koulayev, 2010; De los Santos et al., forthcoming) or switching costs (e.g. Shy, 2002; Kim et al., 2003; Shcherbakov, 2009). The closest of these to our empirical application is the much-cited work by Shy (2002). Unlike the other papers which use more general estimation methodologies, Shy offers a ‘quick and easy’ method for calculating the level of switching costs using only limited data on prices and market shares. In a similar spirit, our empirical application offers a ‘quick and easy’ method that uses only aggregate consumer survey data. However, our method is able to calculate measures for *both* search costs and switching costs.

In the previous literature, only Honka (2010) has simultaneously estimated the level of search costs and switching costs.^7^ Using data on individual consumers' past supplier, search behaviour and final choice of supplier, Honka estimates the actual levels of the two costs in the US auto insurance market. Her estimation methodology differs to ours and is far more general. Indeed, like Shy (2002) our methodology relies on some restrictive underlying assumptions. However, our method is simple to use and requires only minimal data. It is hoped that further work may build on our current strategy to provide a richer estimation methodology that could be used as a practical measurement tool for competition authorities.

## 3 Definitions and distinctions

While previous papers appear to agree on the differences between search costs and switching costs, this section attempts to offer a more formal distinction between the two costs. Farrell and Klemperer (2007, p. 1977) suggest ‘a consumer faces a switching cost between sellers when an investment specific to his current seller must be duplicated for a new seller’. Search costs could appear consistent with this definition. Indeed, in order to change suppliers, a consumer must first incur search costs in order to find and/or process some necessary information about an alternative supplier. However, it is this informational role that produces the key difference between search costs and switching costs. Specifically, it leads to five distinctions. While these distinctions could be viewed as arbitrary, care is later taken to demonstrate the importance of each distinction on each of our results. First, unlike switching costs, search costs cannot be incurred by a consumer who is already fully informed (Distinction 1). Second, a consumer is less informed when making a decision to incur search costs than when making a decision to incur switching costs (Distinction 2). Third, unlike switching costs, search costs can be incurred without then choosing to switch suppliers. That is, expenditure on search costs is a necessary, but not a sufficient, condition for switching suppliers (Distinction 3). Fourth, this then implies that, unlike switching costs, a consumer can incur search costs more than once by searching across multiple firms (Distinction 4). And finally, in a dynamic context, unlike switching costs that are only active after an initial market purchase, search costs may be incurred both pre- and post-purchase (Distinction 5). For the purposes of this paper, the two costs can therefore be defined as follows.

Search costs are the costs incurred by a consumer in identifying a firm's product and price, regardless of whether the consumer then buys the product from the searched firm or not.

Switching costs are the costs incurred by a consumer in changing suppliers that do not act to improve the consumer's pre-purchase information.

Finally, while it is possible that firms may be able influence the level of search costs and switching costs in practice, our later model will follow the tradition within the literatures by treating the two costs as exogenous. Like other standard models, this implies that the costs of switching are best interpreted as exogenous forms of real social costs rather than as contractual costs (Farrell and Klemperer, 2007). For example, switching costs are best considered as those arising from additional effort, service disruption or reduced compatibility, rather than from cancellation fees or lost loyalty benefits.

## 4 Model

Let there be n≥2 firms that each sell a single good with zero production costs. A unit mass of consumers have a zero outside option and each possess a unit demand for the market good. With quasi-linear preferences, let consumer *m* gain an indirect utility (excluding any search or switching costs) of $u_{\mathrm{mi}}=\varepsilon_{\mathrm{mi}}-p_{i}$ if she chooses to buy from firm *i* at price $p_{i}$, where her ‘match value’, $\varepsilon_{\mathrm{mi}}$, is an independent draw from a distribution G(ε) with positive density g(ε) on $\left[\underset{̲}{\varepsilon },\overset{¯}{\varepsilon }\right]$, where $0\leq \underset{̲}{\varepsilon }<\overset{¯}{\varepsilon }$.

The market is assumed to be mature in the sense that each consumer is already partially locked-in to their ‘local’ firm. Each consumer is free to search and trade with their local firm but faces costs of searching and switching in regard to any other ‘non-local’ firm. In line with standard search models (e.g. Stahl, 1989), the main model focuses on a symmetric configuration such that (1/n) consumers are ‘local’ to each firm.^8^ More formally, if consumer *m* is local to firm *i*, she can learn her match value and the price at firm *i*, $\{\varepsilon_{\mathrm{mi}}$, $p_{i}\}$, at zero cost and is also free to trade with firm *i*. However, in order to switch to any non-local firm j≠i, she must first incur c≥0 to learn her match value at firm *j* and firm *j*'s price, $\{\varepsilon_{\mathrm{mj}}$, $p_{j}\}$, and then further incur s≥0 if she still wishes to trade with firm *j*. Search is assumed to be sequential with costless recall. Hence, consumer *m* is able to search any number of non-local firms one by one, incurring a cost of *c* each time, before choosing whether to purchase from her local firm *i* or, for an extra cost of *s*, from a searched non-local firm j≠i.

A one-shot static game is considered where the players select the following strategies simultaneously. Firms each select a single price, $p_{i}$.^9^ At the same time, consumers form conjectures about the firms' pricing strategies and select their ‘search to switch’ strategies. A ‘search to switch’ strategy must prescribe the extent to which the market will be searched, which firms will be searched and which firm, if any, the consumer will trade with. As consumers will consider all non-local firms as identical ex ante in any symmetric price equilibrium, they will remain indifferent over the choice of which non-local firm(s) to search. Consequently, after observing their local firm's offer, $\{\varepsilon_{\mathrm{mi}}$, $p_{i}\}$, a ‘search to switch’ strategy will only need to prescribe whether consumer *m* should start searching beyond her local firm (Step 1), when to stop searching amongst non-local firms (Step 2) and which firm to then trade with (Step 3).

## 5 Equilibrium analysis

### 5.1 Optimal search to switch strategies

This section begins the equilibrium analysis by considering the optimal search to switch strategy for a given consumer. For simplicity, we will assume that the consumer correctly conjectures that all her non-local firms set a price equal to the symmetric equilibrium price, $p^{⁎}$, such that $p_{j}^{e}=p_{j}=p^{⁎}\;\forall j\neq i$. However, in order to help analyse firms' subsequent pricing decisions, no restrictions are placed on the price of the consumer's local firm, $p_{i}$.

To ease the exposition, it is first worth recalling a well-known feature of the optimal strategy in the standard case without switching costs. In particular, suppose a consumer has previously searched a number of non-local firms and that her highest discovered non-local offer, $\left(\varepsilon -p^{⁎}\right)$, exceeds both her outside option and her original local offer, $\max\{0,\varepsilon_{i}-p_{i}\}$. Kohn and Shavell (1974) demonstrate that the consumer should then continue to search amongst any remaining unsearched non-local firms only if her highest previously discovered match value, ε, is less than a threshold level or ‘reservation utility’, x^, as defined in Definition 1.

Definition 1 The reservation utility, x^, is the unique value of *x* that solves $c=\int_{x}^{\overset{¯}{\varepsilon }}(\varepsilon -x)g(\varepsilon )\;d\varepsilon$.^10^

The derivation of this stopping rule is surprisingly simple. First, suppose that the number of remaining unsearched non-local firms, β, equals one. The decision to further search then reduces to a comparison between the highest existing offer, $\left(\varepsilon -p^{⁎}\right)$, and the net benefits of conducting a single search, where for a cost of *c*, a new offer of $\left(\varepsilon \prime -p^{⁎}\right)$ can be discovered. If ε′>ε, this new offer will be preferred to the existing offer. However, if ε′≤ε, the consumer will optimally use her free recall to maintain the existing offer. Using the notation x≡ε for convenience, the consumer will therefore be indifferent over conducting the single search when $(\varepsilon -p^{⁎})=-c+\int_{x}^{\overset{¯}{\varepsilon }}(\varepsilon \prime -p^{⁎})g(\varepsilon \prime )\;d\varepsilon \prime +\int_{\underset{̲}{\varepsilon }}^{x}(\varepsilon -p^{⁎})g(\varepsilon \prime )\;d\varepsilon \prime$. Through simplification, this expression reduces to that used in the definition for the reservation utility. On finding a match value lower (higher) than this reservation utility, it follows that further search will be strictly optimal (suboptimal). One can then use an inductive argument to show that this stopping rule is indeed optimal more generally for any larger number of remaining firms, β≥1.^11^

Lemma 1 will now show how the logic of this strategy can be extended to allow for positive switching costs. Intuitively, the existence of switching costs implies that the decision of whether to further search will now depend upon whether the highest discovered offer originates from a local or a non-local firm. In particular, Lemma 1 demonstrates that the consumer should now employ two different reservation utilities. The consumer should first decide whether to search beyond her local firm in Step 1 by comparing her local offer to a ‘local’ reservation utility, x^−s. If the consumer decides to search, she should then choose whether to further search amongst the non-local firms in Step 2 by comparing her best existing match value with a second reservation utility, x^. Finally, after having chosen to stop searching or having searched the entire market without stopping, the consumer will have discovered some number, J∈[0,n−1], of non-local firm offers (indexed by subscript j). The decision of which searched firm to trade with in Step 3 is trivial. The consumer should trade with the firm offering the best deal net of switching costs, $b=\max\{\varepsilon_{i}-p_{i},\varepsilon_{j}-p^{⁎}-s\}\;\forall j\neq i$, as long as such a deal is preferred to the outside option of zero.^12^

Lemma 1 *Given a search cost*, *c*, *and switching cost*, *s*, *the optimal search to switch strategy consists of the following*.

*Step*1: *Refrain from searching any non-local firm if* max{0,εi−pi}+p⁎≥x^−s, (*or* x^−s<ε̲). *Otherwise search any unsearched non-local firm*.

*Step*2: *Stop searching amongst the non-local firms only if some firm j is found such that* εj≥x^ *or if all firms have been searched*.

*Step*3: *Having stopped searching*, *trade with the searched firm offering the best deal*, $b=\max\{\varepsilon_{i}-p_{i},\varepsilon_{j}-p^{⁎}-s\}\;\forall j\neq i$, *iff* b>0. *Otherwise take the zero outside option*.

The derivation of Steps 1 and 2 is now discussed in detail. However, a consideration of their implications and the roles played by Distinctions 1–4 is best left until we cover the comparative statics of the full model in Section 6.

First, consider Step 2. Having started a non-local search, Lemma 1 suggests that the consumer should stop searching on the discovery of a non-local match value, ε, that is greater than or equal to the reservation utility, x^. This implies that the marginal decision to stop searching amongst non-local firms is, in fact, independent of the level of switching costs and equivalent to that discussed above for the standard case without switching costs. To understand why, consider a consumer who has discovered such a non-local match value ε≥x^. Now, in order to have reached Step 2, the consumer must have decided to initiate a non-local search in Step 1, which required the consumer to have received a sufficiently low local offer, max{0,εi−pi}<x^−s−p⁎. Consequently, the discovered match value, ε≥x^, must yield an offer, $\left(\varepsilon -p^{⁎}-s\right)$, that necessarily dominates the consumer's local offer and outside option, $\max\{0,\varepsilon_{i}-p_{i}\}$. Whether the consumer makes further non-local searches or not, she now knows that she will never return to take her local offer or outside option and that instead, she will definitely trade with this or some other non-local firm. This implies that the consumer will definitely incur switching costs and so the level of switching costs becomes irrelevant in her marginal decision to further search. More formally, if the number of remaining unsearched non-local firms, β, equals one, the consumer will be indifferent over whether to search the remaining non-local firm in order to discover some offer, $\left(\varepsilon \prime -p^{⁎}-s\right)$, when $(\varepsilon -p^{⁎}-s)=-c+\int_{x}^{\overset{¯}{\varepsilon }}(\varepsilon \prime -p^{⁎}-s)g(\varepsilon \prime )\;d\varepsilon \prime +\int_{\underset{̲}{\varepsilon }}^{x}(\varepsilon -p^{⁎}-s)g(\varepsilon \prime )\;d\varepsilon \prime$. The level of switching costs, *s*, then drops out and the expression reduces to the same reservation utility, x^, as that described in Definition 1. The consumer should then optimally stop searching only on the discovery of a match value, ε≥x^, and otherwise keep searching. This logic can then be extended for β≥1 using the inductive arguments described previously.

Let us now move back to the original decision of whether to initiate a non-local search in Step 1. In contrast to Step 2, switching costs now become important because the consumer must compare between (i) collecting the existing local offer (or outside option) which excludes switching costs, $\max\{0,\varepsilon_{i}-p_{i}\}$, and (ii) searching to discover some non-local offer(s) which includes switching costs. Lemma 1 suggests that the consumer should not initiate a non-local search if her local offer (or outside option), normalised for the expected difference between local and non-local prices, $\max\{0,\varepsilon_{i}-p_{i}\}+p^{⁎}$, is greater than or equal to a ‘local’ reservation utility, x^−s. To understand why, first suppose that β=1 such that there is only one (unsearched) non-local firm. If the consumer decides to initiate search she will incur *c* in order to discover a single offer, denoted by $\left(\varepsilon_{1}-p^{⁎}-s\right)$. This new offer will only improve upon the local offer (and outside option) if $\varepsilon_{1}>\max\{0,\varepsilon_{i}-p_{i}\}+p^{⁎}+s$. Denote $x_{1}\equiv \max\{0,\varepsilon_{i}-p_{i}\}+p^{⁎}+s$. The consumer will then be indifferent when $\max\{0,\varepsilon_{i}-p_{i}\}=-c+\int_{x_{1}}^{\overset{¯}{\varepsilon }}(\varepsilon_{1}-p^{⁎}-s)g(\varepsilon_{1})\;d\varepsilon_{1}+\int_{\underset{̲}{\varepsilon }}^{x_{1}}\max\{0,\varepsilon_{i}-p_{i}\}g(\varepsilon_{1})\;d\varepsilon_{1}$. On simplification this condition becomes $c=\int_{x_{1}}^{\overset{¯}{\varepsilon }}(\varepsilon -x_{1})g(\varepsilon )\;d\varepsilon$ and provides an expression for the local reservation utility, x^1. It will then be optimal to refrain from searching whenever x1≡max{0,εi−pi}+p⁎+s≥x^1. However, as the expression for x^1 is identical to that used for x^ in Definition 1, this stopping rule can be re-stated to suggest that search is optimal whenever max{0,εi−pi}+p⁎≥x^−s, as in Lemma 1.^13^ Although slightly more complicated, similar inductive arguments to those used previously can then be employed to show the optimality of this step for β≥1.

### 5.2 Equilibrium pricing decisions

This section considers the firms' optimal pricing decisions. As adopted in other recent search models (e.g. Armstrong et al., 2009), attention is now focused on the uniform distribution to improve tractability and ease interpretation; $G(\varepsilon )=(\varepsilon -\underset{̲}{\varepsilon })/(\overset{¯}{\varepsilon }-\underset{̲}{\varepsilon })$ and $g(\varepsilon )=1/(\overset{¯}{\varepsilon }-\underset{̲}{\varepsilon })$. From Definition 1, x^ then reduces to the following equation:

(1) x^=ε¯−2c(ε¯−ε̲)

From Lemma 1, one can first note that no consumer will wish to search in a symmetric price equilibrium (where $p_{i}=p^{⁎})$ when max{ε̲,p⁎}≥x^−s. Without any consumer search, the firms will be able to sustain the monopoly price. To avoid this less interesting possibility, we concentrate on the case where some positive fraction of consumers do search beyond their local firm in equilibrium. This is ensured by Condition 1.

Condition 1 *In equilibrium*, max{ε̲,p⁎}<x^−s.

Under this condition, one can establish the equilibrium price by first deriving the residual demand for firm *i*. Given a price of $p_{i}$ for firm *i* and a price of $p^{⁎}$ for all other firms, the residual demand for firm *i*, $D_{i}(p_{i},p^{⁎})$, is the sum of four components, as denoted in (2). We will now discuss each component in turn for the case where the difference between $p_{i}$ and $p^{⁎}$ is small^14^ :

(2) $$D_{i}(p_{i},p^{⁎})=F_{\mathrm{Li}}(p_{i},p^{⁎})+F_{\mathrm{NLi}}(p_{i},p^{⁎})+R_{\mathrm{Li}}(p_{i},p^{⁎})+R_{\mathrm{NLi}}(p_{i},p^{⁎})$$

Firm *i*'s local fresh demand, $F_{\mathrm{Li}}(p_{i},p^{⁎})$, is described by (3). It derives from firm *i*'s (1/n) local consumers who choose to buy without searching elsewhere. Any given local consumer chooses not to make a non-local search if max{0,εi−pi}+p⁎≥x^−s and then opts to buy from firm *i* if $\varepsilon_{i}-p_{i}>0$. From Condition 1, the first requirement always ensures the second, and so such local consumers buy with Pr(εi>x^−s+pi−p⁎)=1−G(x^−s+pi−p⁎).

(3) FLi(pi,p⁎)=(1/n)[1−G(x^−s+pi−p⁎)]

Firm *i*'s non-local fresh demand, $F_{\mathrm{NLi}}(p_{i},p^{⁎})$, is presented in (4). It stems from the consumers who are not local to firm *i* but choose to visit firm *i* during their search process and find it optimal to stop and buy. The total number of visits to firm *i* made by non-local consumers can be expressed as (1/n)[G(x^−s)+G(x^−s)G(x^)+⋯+G(x^−s)G(x^)n−2]=(G(x^−s)/n)·∑k=0n−2G(x^)k. The probability of optimally stopping at firm *i* conditional on visiting, equals Pr(εi>x^+pi−p⁎). It is then trivial to show that having stopped at firm *i* it will then be optimal to buy from firm *i*:

(4) FNLi(pi,p⁎)=G(x^−s)n∑k=0n−2G(x^)k·(1−G(x^+pi−p⁎))

In contrast to these two components of fresh demand, firm *i*'s return demand consists of consumers who start searching but never find an offer worth stopping for. They end up searching the entire market before then realising that firm *i* offered the best deal and returning to buy from firm *i*. Depending upon whether such consumers are local or non-local to firm *i*, this demand is denoted as local return demand, $R_{\mathrm{Li}}(.)$, as expressed in (5), or non-local return demand, $R_{\mathrm{NLi}}(.)$, as described in (6). The formal derivation of these two terms is more complicated and is contained within Appendix A.

(5) RLi(pi,p⁎)=1n(ε¯−ε̲)∫max{ε̲,pi}+p⁎−pi+sx^G(ε)n−1dε

(6) RNLi(pi,p⁎)=(n−1)n(ε¯−ε̲)∫max{ε̲,p⁎}+sx^G(ε)n−2G(ε−s)dε

Given the residual demand function presented in (2)–(6), one can now explore the firms' optimal pricing decisions using the standard FOC, $p^{⁎}=-D_{i}(p^{⁎},p^{⁎})/D_{i}^{\prime }(p^{⁎},p^{⁎})$, under the assumption of no profitable large price deviations. Ruling out such deviations to ensure equilibrium existence is hard to fully demonstrate in models such as these (see Appendix B for a detailed discussion). However, Proposition 1 suggests that when it exists, the unique equilibrium price breaks down into two possible cases, depending on the relative magnitude of $p^{⁎}$ and $\underset{̲}{\varepsilon }$. (Throughout the paper, all omitted proofs are contained in Appendix C.)

Proposition 1 *When a symmetric equilibrium exists*, *the equilibrium price is unique and is characterised by* (7), *where* $I(p^{⁎}\leq \underset{̲}{\varepsilon })=1$ *iff* $p^{⁎}\leq \underset{̲}{\varepsilon }$ *and zero otherwise*:

(7) p⁎=1−G(max{ε̲,p⁎})G(max{ε̲,p⁎}+s)n−1(ε¯−ε̲)−1[1+G(x^−s)∑k=0n−2G(x^)k−I(p⁎≤ε̲)G(ε̲+s)n−1]

In the first case, when $p^{⁎}\leq \underset{̲}{\varepsilon }$, all consumers will, at least, be willing to purchase from their local firm and the market will be covered. Each firm will then have an equilibrium demand, $D_{i}(p^{⁎},p^{⁎})$, equal to (1/n) and the price under market coverage, $p_{C}^{⁎}$, will reduce to (8). Note that as market frictions tend to zero, such that x^ and x^−s tend to $\overset{¯}{\varepsilon }$, the equilibrium price converges to $(\overset{¯}{\varepsilon }-\underset{̲}{\varepsilon })/n$. This price corresponds to that found in Perloff and Salop (1985) and reflects the market power that derives purely from product differentiation:

(8) pC⁎=1(ε¯−ε̲)−1[1+G(x^−s)∑k=0n−2G(x^)k−G(ε̲+s)n−1]

In the second case, where $p^{⁎}>\underset{̲}{\varepsilon }$, some consumers will fail to find any offer worthy of purchase and the market can no longer be covered. Having received a low local offer, a consumer will always begin to search and continue to search until finding an attractive offer via Condition 1. However, even after searching the entire market, a consumer will not want to purchase from any of the firms with probability, $\mathrm{Pr}(\varepsilon <p^{⁎})\mathrm{Pr}{\left(\varepsilon <p^{⁎}+s\right)}^{n-1}$, such that each firm's equilibrium demand now reduces to $\left(1/n)[1-G(p^{⁎})G{\left(p^{⁎}+s\right)}^{n-1}\right]$. An explicit expression for the resulting equilibrium price, $p_{\mathrm{NC}}^{⁎}$, is hard to obtain, but the expression for the equilibrium price in (7) now collapses to the following equation:

(9) pNC⁎=1−G(pNC⁎)G(pNC⁎+s)n−1(ε¯−ε̲)−1[1+G(x^−s)∑k=0n−2G(x^)k]

Finally, before examining the comparative statics in detail, it is worth considering some special cases. First, if one sets switching costs to zero, the price derived in (7) offers an original unification of the equilibrium prices found in the search models of Anderson and Renault (1999) and Wolinsky (1986) that assume market coverage and non-market coverage, respectively. Second, by setting search costs to zero, such that x^=ε¯, the model collapses to a static analysis of switching costs which shares some similar features to that used in some recent dynamic studies, such as Cabral (2008) and Dubé et al. (2009).

## 6 Comparative statics

In this section, we examine the relative mechanisms by which changes in the level of search costs and switching costs affect the equilibrium price and welfare. Sections 6.1 and 6.2 consider how the costs affect the equilibrium price under non-market coverage and market coverage, respectively. Condition 1 is maintained throughout. Without it, firms can sustain the monopoly price and increases in either cost will have no effect on the equilibrium price. Finally, Section 6.3 analyses the effects on welfare.

### 6.1 Non-market coverage

The case of non-market coverage is easiest to analyse. As a preliminary step, Proposition 2 first confirms that higher levels of either friction prompt an increase in the equilibrium price.^15^

Proposition 2 *The equilibrium price under non-market coverage*, $p_{\mathrm{NC}}^{⁎}$, *is increasing in the level of search costs*, *c*, *for all* n≥2 *and increasing in the level of switching costs*, *s*, *for all* n>2, *and for* n=2 *if* $\underset{̲}{\varepsilon }>0$.

Of more interest are the different underlying mechanisms that generate such price effects. Proposition 3 now suggests that the mechanisms by which search costs and switching costs affect competition are so different that their effects on the equilibrium price can consistently differ in magnitude.

Proposition 3 *Under non-market coverage*, *the marginal effect from an increase in search costs on the equilibrium price is always larger than the marginal effect from an increase in switching costs*.

To gain an understanding of Proposition 3, note that the sign of $\left(\partial p_{\mathrm{NC}}^{⁎}/\partial c)-(\partial p_{\mathrm{NC}}^{⁎}/\partial s\right)$ depends upon the sign of the sum of the three expressions presented below. One can use the (static) Distinctions 1–4 to then provide an intuition for each expression and show that each expression is positive, such that search costs always have the consistently larger marginal effect:

−pNC⁎(ε¯−ε̲)G(x^−s)∂∑k=0n−2G(x^)k∂c

−pNC⁎(ε¯−ε̲)∑k=0n−2G(x^)k∂G(x^−s)∂c−∂G(x^−s)∂s

(10) $$-\frac{\partial (1-G(p_{\mathrm{NC}}^{⁎})G{\left(p_{\mathrm{NC}}^{⁎}+s\right)}^{n-1})}{\partial s}$$

The first expression relates to the effect of search costs on increasing the equilibrium price by reducing the extensiveness of consumers' non-local search activity. Holding constant the fraction of consumers who start searching, G(x^−s), an increase in the cost of search prompts such consumers to search fewer non-local firms by decreasing the reservation utility, ∂x^/∂c<0. No such effect is created by an increase in switching costs because we know from Lemma 1 that the marginal decision to make a further non-local search, via x^, is independent of *s*. This follows from the assumption that only search costs can be incurred across multiple suppliers (Distinction 4).

The second expression concerns the net impact of search costs relative to switching costs on increasing the equilibrium price by deterring consumers from initiating any non-local search activity. Holding constant the extensiveness of any search, ∑k=0n−2G(x^)k, a rise in either cost reduces the fraction of consumers who actually begin to search, ∂G(x^−s)/∂c<0 and ∂G(x^−s)/∂s<0. However, one can show that a unit rise in search costs deters consumers from initiating a non-local search by more than a unit increase in switching costs, ∂G(x^−s)/∂c<∂G(x^−s)/∂s<0. This difference is key and relates to a combination of Distinctions 2 and 3. To best understand it, recall the expression for the net benefits of a first non-local search, $-c+\int_{x_{1}}^{\overset{¯}{\varepsilon }}(\varepsilon_{1}-p^{⁎}-s)g(\varepsilon_{1})\;d\varepsilon_{1}+\int_{\underset{̲}{\varepsilon }}^{x_{1}}\max\{0,\varepsilon_{i}-p_{i}\}g(\varepsilon_{1})\;d\varepsilon_{1}$. Within this expression, first note that the consumer expects to incur search costs with probability one, regardless of whether or not the discovered non-local offer is attractive. This derives from the fact that the decision to incur search costs must be made when the consumer is relatively uninformed (Distinction 2). Second, note, in contrast, that the consumer expects to incur switching costs only if the discovered offer is attractive, which occurs with a probability less than one. This stems from the fact that the consumer is able to decide not to switch after searching (Distinction 3). Hence, in evaluating whether or not to initiate a non-local search, the consumer places a greater per-unit weight on search costs rather than on switching costs, and this makes them particularly powerful in deterring non-local search activity and generating market power.

Finally, the last expression relates to the effect of an increase in switching costs on consumers that have searched the entire market. An increase in switching costs makes such fully informed consumers less likely to switch to a non-local firm and here, under non-market coverage, more likely to leave the market without making a purchase. This reduces each firm's demand, $D_{i}(p_{\mathrm{NC}}^{⁎},p_{\mathrm{NC}}^{⁎})=(1/n)[1-G(p^{⁎})G{\left(p^{⁎}+s\right)}^{n-1}]$, and provides an incentive for each firm to actually lower their price, as $p^{⁎}=-D_{i}(.)/D_{i}^{\prime }(.)$, which further enhances the difference between $\partial p_{\mathrm{NC}}^{⁎}/\partial c$ and $\partial p_{\mathrm{NC}}^{⁎}/\partial s$.^16^ An equivalent effect for search costs does not exist due to the assumption that only switching costs are active when a consumer is fully informed (Distinction 1).

As a simplified summary, we can see that the relative power of search costs largely derives from the following. A consumer who searches beyond her local firm (i) must incur a search cost and yet may not choose to later incur a switching cost (via Distinctions 2 and 3) and (ii) may choose to incur multiple search costs (via Distinction 4). Many remaining results in the paper will build on this foundation intuition.

### 6.2 Market coverage

We now move on to the setting of market coverage. The mechanisms by which search and switching costs affect the equilibrium price are very similar to those under non-market coverage. Consequently, it can be shown again that the equilibrium price is increasing in each friction.

Proposition 4 *The equilibrium price under market coverage*, $p_{C}^{⁎}$, *is increasing in both the level of search costs*, *c*, *and the level of switching costs*, *s*, *for all* n≥2.

However, there is one difference in the mechanism regarding the effect of switching costs on deterring fully informed consumers from trading with non-local firms. Under non-market coverage, this made such consumers more likely to exit the market and gave firms an incentive to decrease their price. Now, under market coverage, this makes such consumers more inclined to purchase from their local firm and gives firms an incentive to *increase* their price. This change in mechanism makes any difference in magnitude between the marginal effects of search costs and switching costs less stark. However, Proposition 5 shows that search costs will continue to have the larger marginal effect on price if the number of firms, *n*, is sufficiently large.

Proposition 5 *Under market coverage*, *the marginal effect of search costs on the equilibrium price is always larger than the marginal effect of switching costs when the number of firms is greater than or equal to four*.

When there are two or three firms, either cost can have the larger marginal effect. However, when the number of firms is larger, search costs have the consistently bigger impact on market power. Intuitively, this follows from two effects. First, as the number of options grows, consumers that have searched the entire market become more price sensitive and are less affected by the loyalty-inducing effects of switching costs. Second, an increase in the number of firms tilts the relative composition of each firms' demand towards non-local consumers and enhances the effects of search costs on deterring the initiation and extensiveness of any search activity.

### 6.3 Welfare

In most cases, search costs appear to be the relatively more powerful determinant of market power. To be able to provide better policy advice, this subsection considers their relative effects on welfare. We now state Proposition 6, which applies to both the cases of non-market coverage and market coverage.

Proposition 6 *If the marginal effect from an increase in search costs on price is larger than the marginal effect from an increase in switching costs*, $\partial p^{⁎}/\partial c>\partial p^{⁎}/\partial s$, *then*, *relative to a unit increase in switching costs*, *a unit increase in search costs generates a greater reduction in aggregate consumer surplus*, *CS*, *a greater decrease in total welfare*, *W*, *and a greater increase in industry profits*, Π.

Proposition 6 can be explained as follows. First, consider industry profits, defined as $\Pi =p^{⁎}D(p^{⁎},p^{⁎})$ where $D(p^{⁎},p^{⁎})=(1-G(\max\{\underset{̲}{\varepsilon },p^{⁎}\}))G{\left(\max\{\underset{̲}{\varepsilon },p^{⁎}\}+s\right)}^{n-1}$. If switching costs have the relatively weaker effect on the equilibrium price then they must also have the relatively weaker effect on industry profits because, unlike an increase in search costs which leaves the market size unchanged, $\partial D(p^{⁎},p^{⁎})/\partial c=0$, an increase in switching costs can also reduce the size of the market, $\partial D(p^{⁎},p^{⁎})/\partial s\leq 0$.

The effects on aggregate consumer surplus are more complex. Consider a marginal increase in switching costs. This will affect consumer surplus *directly* in two ways. It will (i) increase the price paid by existing buyers and (ii) increase the cost of switching to existing switchers. If one denotes the number of existing switchers as $K_{s}$ such that the aggregate cost of switching across all switchers is ${\mathrm{sK}}_{s}$, then a marginal increase in switching costs will reduce aggregate consumer surplus directly by an amount, $D(p^{⁎},p^{⁎})\cdot (\partial p^{⁎}/\partial s)+K_{s}$. Further, by using the results of Sections 6.1 and 6.2, we also know that an increase in switching costs will affect aggregate consumer surplus *indirectly* by changing consumers' behaviour. In particular, while an increase in switching costs will have no effect on the extensiveness of consumers' search activity, it will (iii) decrease the number of consumers who choose to search beyond their local firm, via a reduction in the local reservation utility, x^−s, and (iv) deter some consumers that have searched the entire market from switching to a non-local supplier.

With the application of an envelope-style argument, related to that used in Armstrong et al's (2009, Proposition 3) search model, we now claim that effects (iii) and (iv) have no (first-order) impact. Consider effect (iii). For a small increase in switching costs to prompt a consumer to stop searching beyond her local firm, the consumer must have been previously on the decision margin – being exactly indifferent between the payoffs from buying from her local firm without searching and the expected payoffs from initiating a non-local search – otherwise the consumer would continue to search. Hence, on average, an increase in switching costs will have no (first-order) impact on such a consumer's surplus. Similarly, in effect (iv), for a small increase in switching costs to prompt a fully informed consumer to refrain from switching to a non-local supplier, the consumer must have been previously indifferent between the payoffs from switching and the payoffs from exiting the market or returning to her local firm. This leaves only the direct effects (i) and (ii) and allows us to state that $\partial \mathrm{CS}/\partial s=-D(p^{⁎},p^{⁎})[\partial p^{⁎}/\partial s]-K_{s}$.

A similar argument can be used in respect to an increase in search costs. By denoting $K_{c}$ as the aggregate number of non-local searches made by all consumers, it follows that $\partial \mathrm{CS}/\partial c=-D(p^{⁎},p^{⁎})[\partial p^{⁎}/\partial c]-K_{c}$. The only direct effects from an increase in search costs on aggregate consumer surplus are an increase in price for existing buyers and an increase in the cost of each existing search. There is no (first-order) impact from other effects that prompt some consumers to make fewer searches because these consumers will have previously been on a decision margin.

This implies that $\left(\partial \mathrm{CS}/\partial c)-(\partial \mathrm{CS}/\partial s)=-D(p^{⁎},p^{⁎})[\partial p^{⁎}/\partial c-\partial p^{⁎}/\partial s]-(K_{c}-K_{s}\right)$. Hence, search costs will definitely provide the relatively larger marginal effect on aggregate consumer surplus whenever $\partial p^{⁎}/\partial c>\partial p^{⁎}/\partial s$, if the total number of non-local searches, $K_{c}$, is equal to or larger than the total number of switches, $K_{s}$. The proof in the appendix demonstrates that this is always true in equilibrium. The reasoning is simple. $K_{c}$ is always strictly larger than $K_{s}$ due to a combination of Distinctions 2–4 – not all searching consumers may switch and/or some switching consumers may search more than one firm.

Finally, one can use similar arguments to show that search costs also provide the relatively larger marginal effect on total welfare. As the loss in consumer surplus from an increased price for existing buyers is appropriated by firms in industry profits, the only (first-order) effects on total welfare stem from the increased cost of existing search and switching activity, such that $\partial W/\partial c=-K_{c}$ and $\partial W/\partial s=-K_{s}$. Proposition 6 then follows from above as $K_{c}>K_{s}$.

To summarise, Propositions 2– 6 suggest that in most cases, search costs have the more powerful marginal effect of market power, profits, consumer surplus and total welfare. These results have practical implications. First, government authorities may wish to focus their limited resources on reducing search costs rather than switching costs. Regardless of the levels of the two costs, our results suggest that the benefits from a unit reduction in search costs will often outweigh the benefits from a unit reduction in switching costs. While the authorities' optimal decision will also depend upon the associated resource costs of each policy intervention, this implies that authorities may prefer to improve, say, the provision of consumer information rather than legislating to ease the switching process. Second, the results also suggest that industries that wish to (collusively) increase market profits may prefer to focus their attempts on increasing market-level search costs rather than switching costs. Under this logic, industry agreements to curb levels of informative advertising may appear particularly potent. Competition authorities should be watchful for such strategies.

## 7 Extensions

This section now investigates the robustness of the results by presenting a variety of extensions. Most importantly, we first consider the introduction of dynamic effects. We then investigate the possibility of costly local search, asymmetric consumer locations, price discrimination and switching without searching.

### 7.1 Dynamic effects

In order to provide a clear analysis of the subtle differences between the two frictions, the model has deliberately neglected any consideration of dynamic competition. However, we expect that it would only strengthen the findings. To illustrate, consider a two-period version of the model where each consumer enters the market in period 1 with no switching costs, makes an initial purchase at some firm and then finds it costly to switch away from that firm in period 2. In effect, our model has only analysed period 2, where firms have an incentive to exploit or ‘harvest’ their locked-in (local) consumers. Now consider period 1. If the firms are myopic and do not consider the impact of their period 1 decisions on their period 2 profits, then it is clear that the comparative statics would remain consistent with our original model. If, instead, the firms are forward-looking, then the comparative statics are likely to be strengthened. To understand why, note that the introduction of dynamic competition often erodes the anti-competitiveness of switching costs by prompting firms to lower their initial (period 1) prices in order to ‘invest’ in market share and earn higher future ‘lock-in’ profits (in period 2) (Farrell and Klemperer, 2007). However, with the use of Distinction 5 which suggests that search costs are active not just after an initial market purchase (in period 2) but also beforehand when consumers first enter a market (in period 1), we can argue that the anti-competitive effects of search costs will not be eroded in such a way. Unlike switching costs, search costs will create an upward pressure on prices in both period 1 and period 2 and so they will continue to provide the more powerful effects on welfare.

### 7.2 Costly local search

It was originally assumed that consumers could search their local firms without cost. We will no show that the introduction of costly local search makes no difference to the pricing equilibrium and actually strengthens the comparative static results on welfare. If a local search now costs the same as a non-local search, c>0, a consumer's decision to enter the market is no longer trivial. The consumer must choose between (i) staying out of the market to receive the zero outside option, (ii) making a first search to their local firm to discover an offer with expected value, $E(\varepsilon_{i})-p^{⁎}$ and (iii) making a first search to a non-local firm to discover an expected offer, $E(\varepsilon_{j})-p^{⁎}-s$. Option (iii) is dominated. However, if the consumer chooses to search its local firm, the local offer will only be attractive relative to the outside option if $\varepsilon_{i}>p^{⁎}$. By letting $x\equiv p^{⁎}$, we know that the consumer will then be indifferent between options (i) and (ii) when $0=-c+\int_{x}^{\overset{¯}{\varepsilon }}(\varepsilon_{i}-x)g(\varepsilon_{i})\;d\varepsilon_{i}$. The value of *x* that solves this expression, x^, coincides with the definition for the standard reservation utility in Definition 1. Searching the local firm will then be optimal whenever x<x^ or equivalently, when 0<x^−p⁎. However, this condition is always satisfied through Condition 1: max{ε̲,p⁎}<x^−s, and so all consumers will always make a first search to their local firm as observed in the main model. It then follows that the equilibrium pricing equilibrium remains unchanged. However, any increase in the level of search costs will now generate additional reductions in consumer surplus and total welfare by increasing the cost of existing local searches, such that the welfare-damaging effects of search costs are further enhanced relative to switching costs.

### 7.3 Asymmetric consumer locations

As in standard search models, the basic model assumed that each firm was endowed with a symmetric share of local consumers. However, in practice, it may be the case that all consumers are local to the *same* firm. For example, after a monopoly market has been liberalised all consumers may face costs of search and switching away from the incumbent. To show how our results are robust in such a setting, consider an equilibrium where the incumbent, say Firm 1, sets a price, $p_{1}^{⁎}$, and where the (n−1) entrants, with no local consumers, each set some price, $p_{2}^{⁎}$. To offset the additional complexity, we focus on the case where *n* is large. Proposition 7 follows.

Proposition 7 *Consider the incumbent model with* n→∞ *and* c>0. *Then*, *relative to a unit increase in switching costs*, *a unit increase in search costs generates a larger increase in equilibrium prices and industry profits*, *and a greater reduction in consumer surplus and total welfare*.

To understand why the incumbent's price and the entrants' prices are more sensitive to search costs rather than switching costs, note that when *n* is large, any consumer who searches beyond the incumbent will always find an attractive non-local offer and never return to the incumbent. This makes Distinction 1 inactive. Now consider the incumbent's choice of price. Its demand derives solely from consumers that wish to buy without starting a non-local search. From previous results, we know that an increase in search costs provides the relatively stronger effect in deterring consumers from starting such a search via Distinctions 2 and 3. Consequently, an increase in search costs will provide the relatively stronger effect in raising the incumbent's price. To consider the entrants' price, note that each entrant's demand derives solely from consumers that started to search beyond the incumbent and decided to stop after visiting the entrant. A rise in search costs deters consumers from pursuing further non-local searches and allows each entrant to increase its price. However, a rise in switching costs has no such effect on the incentives to further search via Distinction 4 and so leaves entrants' prices unchanged. Finally, having confirmed the relative effects on prices, the relative effects on welfare can also be established using similar arguments to those used in Proposition 6.

### 7.4 Price discrimination

Contrary to the original model, it is sometimes possible for firms to discriminate between their local and non-local consumers. We will now demonstrate that our results remain robust when any firm *i* can set a price, $p_{\mathrm{iL}}$, to its local consumers and a price, $p_{\mathrm{iNL}}$, to its non-local consumers. To offset the added complexity, we again focus on the case where *n* is large. Within this setting, it is also possible to allow a very general configuration of consumer locations, where firm *i* has a proportion of local consumers equal to $a_{i}$, such that $\sum_{i=1}^{n}a_{i}=1$.

Proposition 8 *Consider the price discrimination model with* n→∞ *and* c>0. *Then*, *relative to a unit increase in switching costs*, *a unit increase in search costs generates a larger increase in equilibrium prices and industry profits*, *and a greater reduction in consumer surplus and total welfare*.

The intuition for this result is surprisingly similar to that in Section 7.3. First consider local prices. Given that *n* is large, there is no return demand and so each firm's local demand derives only from consumers that wish to purchase without starting a non-local search. Via Distinctions 2 and 3, a rise in search costs deters consumers from starting to make a non-local search and raises the optimal local price by an amount greater than that generated by a rise in switching costs. Second, consider non-local prices. Each firm's non-local demand derives from consumers that have started a non-local search and then decided to stop. A rise in search costs deters consumers from pursuing further non-local searches and raises the equilibrium non-local price, but an increase in switching costs has no such effect via Distinction 4. Finally, the results on welfare again follow easily using similar arguments to those used in Proposition 6.

### 7.5 Switching without searching

Finally, in the vast majority of industries, it is clear that consumers will necessarily have to incur positive costs in gathering and processing some information before switching suppliers. However, in some cases it may be possible for a consumer to bypass any such activity by blindly switching to an alternative firm without first knowing its price or characteristics. For example, this possibility is discussed in Giulietti et al.'s (2010) study of search costs in the UK electricity market where there are high levels of doorstep selling activity. The basic model can be shown to still apply in such situations provided the level of search costs is sufficiently small as to ensure consumers still find it optimal to search before switching.^17^

## 8 Data application

As a secondary contribution, this final section considers a data application. In the spirit of Shy's (2002) ‘quick and easy’ method for calculating the level of switching costs, we show how one can use some conditions from the consumers' optimal search to switch strategy together with aggregate consumer survey data to recover a set of ‘back of the envelope’ measures for *both* search costs and switching costs. While the model's underlying assumptions are clearly restrictive in a real-world context, the resulting empirical methodology is extremely simple and requires only minimal data. It is hoped that further work may expand the current methodology to improve its generality and enable it to become a practical measurement tool for competition authorities in the future. After presenting the methodology in Section 8.1, we provide an example with data from eight different markets in 8.2 and discuss the methodology's limitations in 8.3.

### 8.1 Methodology

The methodology is able to generate separate estimates for the two costs, c^ and s^, as a proportion of the maximum potential gains available from search, $\left(\overset{¯}{\varepsilon }-\underset{̲}{\varepsilon }\right)$. It makes use of two equilibrium conditions that link observable aggregate consumer behaviour to the underlying levels of search and switching costs. The two conditions hold for any number of firms larger than two and regardless of the market coverage assumption.^18^ First, the model predicts that consumers should search beyond their local firm in equilibrium (where $p_{i}=p^{⁎}$) if they receive a local match value lower than x^−s. Hence, the proportion of consumers who choose to search, denoted by *a*, should be described by the following equation:

(11) a=G(x^−s)

Second, provided there are more than two firms, the model suggests that consumers will switch after making exactly one non-local search if they receive a local match value lower than x^−s and discover a first non-local offer exceeding x^. Hence, the proportion of consumers who choose to switch after only one non-local search, *b*, should be described by the following equation:

(12) b=G(x^−s)[1−G(x^)]

One can now use (11) and (12) to construct the two measures. As a first step, *b* can be divided by *a* to suggest that the fraction of searching consumers who are observed to switch after only one search, (b/a), equals 1−G(x^). Crucially, this relationship is independent of the level of switching costs. This is because it refers to the extensiveness of search, which we know is determined only by the level of search costs. Hence, by using (b/a)=1−G(x^) with the definition for the reservation utility, x^=ε¯−2c(ε¯−ε̲), one can construct a measure for search costs as a simple function of (b/a), (13). Intuitively, as (b/a) rises, the methodology proposes a relatively higher measure of search costs in order to explain why more searching consumers stop after only one search:

(13) c^(ε¯−ε̲)=0.5ba2

Having constructed the measure of search costs for a given (b/a), a separate measure for switching costs can then be derived by substituting x^=ε¯−(ε¯−ε̲)(b/a) into (11) to give (14). Intuitively, the measure derives from the difference between the actual proportion of consumers who do not search beyond their local firm, (1−a)=1−G(x^−s), and the predicted proportion that would not search if there were only search costs, 1−G(x^)=(b/a):

(14) s^(ε¯−ε̲)=(1−a)−ba

To calculate numerical values for the two measures for an actual market, one can then use aggregate survey data about the levels of *a* and *b*. However, the interpretation of the measures will remain limited because the maximum gains from search, $\left(\overset{¯}{\varepsilon }-\underset{̲}{\varepsilon }\right)$, are often unobservable. Therefore, it may be helpful to also calculate the ratio of the two measures in order to obtain a scale-free estimate of the level of switching costs relative to search costs:

(15) s^c^=(1−a)−ba0.5ba2

Finally, it is useful to highlight the importance of accounting for both forms of friction separately within the methodology. To illustrate, suppose a researcher falsely believes that search costs are negligible. The condition in (11) would then imply that any consumer with a local match value lower than $\overset{¯}{\varepsilon }-s$ will search, $a=G(\overset{¯}{\varepsilon }-s)$. This generates a ‘single-cost’ measure for switching costs, s^sing/(ε¯−ε̲)=(1−a). Unfortunately, this measure suffers from an upward bias, (s^sing−s^)/(ε¯−ε̲)=(b/a)>0, because it falsely attributes all the observed inertia in consumers' unwillingness to start searching to switching costs alone. Now consider the parallel case where the researcher falsely believes s=0. The proposed measure for search costs, c^ in (13), remains unbiased because it is based on the extensiveness of search which is independent of the level of switching costs. However, the researcher may now be tempted to use a simpler, alternative measure instead. Using only the proportion of searchers, *a*, the implied condition, a=G(x^), generates a single-cost measure for search costs, c^sing/(ε¯−ε̲)=(1−a)2/2. Yet, this also suffers from an upward bias because it too falsely attributes all the observed consumer inertia to only one of the costs. Due to the possibility of these biases, we conclude that any single-cost study that uses a related identification strategy based on the (un)willingness of consumers to search may offer misleading estimates. To avoid this, future studies should make explicit account for both frictions or use an alternative identification strategy. In the latter regard, measuring search costs with the use of the extensiveness of search appears particularly promising.^19^

### 8.2 An example

As an example, the proposed measures are now calculated for eight different markets from the UK using responses from a survey of 2027 consumers.^20^ If a consumer was active in the specified market, the survey asked (among other questions) (i) whether the consumer had searched for an alternative supplier in the past three years, (ii) whether the consumer had switched suppliers in the past three years, and (iii) if the consumer had switched within the last three years, how many suppliers they searched beforehand. In our benchmark case, we employ the aggregate responses to these questions to provide direct data on the proportions, *a* and *b*. The survey values and the resulting measures are presented in the first five columns of Table 1 . However, this approach assumes that the relevant decision period over which the proportions *a* and *b* should be measured is three years. In some markets, such as the market for mortgages, this may be appropriate.^21^ However, in others, the relevant decision period may be shorter. For a comparison, the next three columns of Table 1 therefore recalculate the measures in an alternative setting with a one-year decision period. These measures are denoted by a subscript 1 and are computed by using $a_{1}=(a/3)$ and $b_{1}=(b/3)$ under the assumption of constant search and switching rates over the three year period. Finally, the last two columns of Table 1 also report the biased single-cost measures, c^sing and s^sing, as defined in the last section (for the benchmark three-year decision period).

Several observations can be made. First, the results suggest that the ratio of switching costs to search costs in the benchmark case, (s^/c^), is extremely high. Switching costs are calculated to be between 230 and 6682 times larger than search costs. Indeed, while search costs are estimated to cover only 0.01–0.3% of the maximum gains from search, switching costs cover 48–73% of the maximum gains available and are large enough to discourage the vast majority of consumers from searching beyond their local firm. From the discussion above, these results derive from the small proportion of searching consumers that switch after only one search, (b/a), which suggests search costs are low, and the very large proportion of consumers that still refrain from any non-local search, *a*, which suggests switching costs are high.

Second, the relative size of switching costs is estimated to be even larger under the assumption of an annual decision period, (s^1/c^1). Intuitively, the estimates of search costs remain unaffected because the fraction of searching consumers who switch after only one search, (b/a), is independent of the chosen decision period. However, the proportion of consumers who search, *a*, is lower under the annual assumption and the model explains this by raising the estimated level of switching costs.

As detailed below, these initial estimates are open to many limitations. However, the finding that switching costs may be so large relative to search costs does raise the possibility that authorities may prefer to lower switching costs rather than search costs. While our previous theoretical results suggested that the marginal benefits from a reduction in search costs are often larger than those from a reduction in switching costs for any levels of the two costs, the overall net welfare effects could be reversed if switching costs are sufficiently easier to reduce. Authorities should bear this point in mind when making their policy decisions.

Finally, one can confirm the potential upward bias of the single-cost measures, c^sing and s^sing, by comparing them with the estimated measures of the two costs, c^ and s^. The bias is very pronounced in regard to the search cost measures because they falsely include the more substantial effects of switching costs.

### 8.3 Limitations

The finding that switching costs are larger than search costs does not seem inconsistent with the existing literature.^22^ However, the fact that we estimate switching costs to be so large relative to search costs is likely to be an indication of the methodology's limitations. Indeed, while the methodology provides a quick and simple method for measuring the two costs separately, it clearly has many restrictive assumptions. It is hoped that further work can build on our initial work to offer a more general estimation methodology for the future.

Some major limitations arise from the assumptions regarding consumers' product valuations. First, the methodology relies heavily on the unrealistic assumption that the product valuations are drawn from a uniform distribution. More substantially, the methodology is also limited by the assumption that the product valuations are independently drawn from an identical distribution. This implies that any excessive reluctance by consumers to search or switch away from a supplier is automatically attributed to the existence of market frictions. However, in practice, such inertia could also be explained by heterogeneous consumer preferences and yet, the methodology offers no account for this. A simple form of this issue can be illustrated as follows. Suppose each consumer places an (unobserved) premium, γ>0, on their local firm *i* such that $u_{i}=\gamma +\varepsilon_{i}-p_{i}$. This has no effect on the extensiveness of any search activity but consumers are now more reluctant to start searching, with a=G(x^−s−γ). As a result, the methodology is then only able to compute $\left(s+\gamma )/(\overset{¯}{\varepsilon }-\underset{̲}{\varepsilon })=(1-a)-(b/a\right)$ and cannot separately identify the level of switching costs, *s*, from the preference parameter, γ.^23^

More sophisticated methodologies are able to distinguish between market frictions and consumer heterogeneity using more detailed data. Typically, in addition to data on firms' prices and possibly, product characteristics, such studies employ further data on individual consumer's choices over time to estimate switching costs (e.g. Shcherbakov, 2009; Dubé et al., 2010) or extra data on consumers' search histories to estimate search costs (e.g. Koulayev, 2010; De los Santos et al., forthcoming). To estimate the two costs simultaneously, Honka (2010) draws on a dataset with both these features. However, like Shy's (2002) single-cost study of switching costs, we are unable to account for consumer heterogeneity because we base our estimates around a static model with (ex ante) identical consumers. Consequently, the current methodology is likely to overestimate the levels of market friction, especially in regard to switching costs.

A further limitation from the use of a static model also arises from the implicit assumption that consumers are myopic. Consumers only take into account current variables when making their decisions, while ignoring any future expected differences between firms. Most empirical studies ignore these effects but Shcherbakov (2009) introduces forward-looking consumers into his dynamic analysis of switching costs and shows that a myopic model would have underestimated the level of switching costs. Incorporating such features into our methodology and into the wider literature would clearly be useful for the future.

## 9 Conclusions

To help better understand and measure market frictions, this paper has offered a unified analysis of search costs and switching costs. In its main contribution, the paper has documented the theoretical mechanisms by which the two frictions can affect competition and welfare. Far from being equivalent, these mechanisms are so different that the effects of the two costs can consistently differ in magnitude. Indeed, the paper has shown that, per unit, search costs are often the more anti-competitive and welfare-damaging. While the optimal policy intervention will also depend upon the associated resource costs, this suggests that, in response to the concerns about market frictions in markets such as those for banking in Europe, the benefits from reducing search costs may outweigh the benefits from reducing switching costs.

As a secondary contribution, the paper has used insights from the theoretical model to construct separate empirical measures for search costs and switching costs. It has also highlighted the importance of accounting for both frictions in empirical work by showing how some ‘single-cost’ measures can exhibit an upward bias.

Overall, it is hoped that the paper may prompt researchers to think further about search costs and switching costs. Empirically, we hope that future work will continue to develop more sophisticated estimation methodologies that account for the existence of both costs. Theoretically, the finding that search costs are often particularly anti-competitive and welfare-damaging underlines the importance of consumer search as an ongoing and increasingly active field of study. Finally, it is hoped that our results could also be extended to help explore the role of search costs and switching costs in labour markets.

## Figures and Tables

**Table 1:** Survey responses and estimated measures of search and switching costs.

Market	*a*	*b*	c^(ε¯−ε̲)	s^(ε¯−ε̲)	s^c^	c^1(ε¯−ε̲)	s^1(ε¯−ε̲)	s^1c^1	c^sing(ε¯−ε̲)	s^sing(ε¯−ε̲)
Electricity	0.306	0.016	0.001	0.642	469	0.001	0.846	619	0.241	0.694
Mobile phone	0.342	0.011	0.001	0.626	1210	0.001	0.854	1651	0.216	0.658
Fixed phone line rental	0.217	0.017	0.003	0.705	230	0.003	0.849	277	0.307	0.783
National and overseas calls	0.253	0.017	0.002	0.680	301	0.002	0.848	376	0.279	0.747
Broadband	0.491	0.016	0.001	0.476	897	0.001	0.804	1514	0.130	0.509
Car insurance	0.493	0.006	0.000	0.495	6682	0.000	0.823	11119	0.129	0.507
Mortgage	0.436	0.008	0.000	0.546	3241	0.000	0.836	4968	0.159	0.564
Current bank account	0.221	0.011	0.001	0.729	589	0.001	0.877	708	0.303	0.779

## Appendix A Derivation of return demand

A consumer who is local to firm *i* will form part of firm *i*'s local return demand, (5), if she (i) starts to search from firm *i*, (ii) chooses to search the entire market without optimally stopping, but then prefers to buy from firm *i* rather than (iii) buying from any other firm or (iv) taking the outside option. This occurs with the probability that (i) εi<x^−s+pi−p⁎, (ii) εj<x^∀j≠i, (iii) $\varepsilon_{i}-p_{i}\geq \varepsilon_{j}-p^{⁎}-s\;\forall j\neq i$ and (iv) $\varepsilon_{i}\geq p_{i}$. As (ii) is non-binding, this probability can be expressed by ∫max{ε̲,pi}x^−s+pi−p⁎G(ε−pi+p⁎+s)n−1g(ε)dε. With simplification, the use of the uniform assumption, and multiplying over firm *i*'s (1/n) local consumers, the firm's local return demand can then be expressed by (1/n(ε¯−ε̲))∫max{ε̲,pi}+p⁎−pi+sx^G(ε)n−1dε.

A consumer who is local to some firm j≠i will form part of firm *i*'s non-local return demand, (6), if she (i) starts to search from firm *j*, and continues to search without stopping at (ii) firm *i* or (iii) any other firm k≠j,i, but then prefers to buy from firm *i* rather than (iv) buying from firm *j*, (v) buying from any firm *k* or (vi) taking the outside option. This occurs with the probability that (i) εj<x^−s, (ii) εi<x^+pi−p⁎, (iii) εk<x^ ∀k≠i,j, (iv) $\varepsilon_{i}-p_{i}-s\geq \varepsilon_{j}-p^{⁎}$, (v) $\varepsilon_{i}-p_{i}-s\geq \varepsilon_{k}-p^{⁎}-s$ ∀k≠i,j and (vi) $\varepsilon_{i}\geq p_{i}+s$. Conditions (i) and (iii) are non-binding. Further, by rewriting (iv) as $\varepsilon_{j}\leq \varepsilon_{i}-p_{i}+p^{⁎}-s$, observe that the probability that condition (iv) is met is zero unless $\varepsilon_{i}\geq \underset{̲}{\varepsilon }+p_{i}-p^{⁎}+s$ and so with this further condition, the total probability can then be expressed by ∫max{pi+s,ε̲+pi−p⁎+s}x^+pi−p⁎G(ε−pi+p⁎−s)G(ε−pi+p⁎)n−2g(ε)dε. With simplification, the use of the uniform assumption and multiplying over the ((n−1)/n) consumers that are not local to firm *i*, firm *i*'s non-local return demand can be expressed by [(n−1)/n][1/(ε¯−ε̲)]∫max{ε̲,p⁎}+sx^G(ε)n−2G(ε−s)dε.

## Appendix B Equilibrium existence

The existence of equilibrium is difficult to fully demonstrate in models such as these due to potential kinks in demand which can prevent profit functions from being globally concave. See Christou and Vettas (2008) for a technical discussion within a related model of informative advertising. Indeed, in our context, the expressions for the components of residual demand, presented in (3)–(6), are only valid if $\left|p_{i}-p^{⁎}\right|$ is not too large. In particular, equations (3)–(6) are only valid for pi∈[pB=p⁎+ε̲−(x^−s),pT=p⁎+ε¯−x^]. If $p_{i}<p_{B}$, none of firm *i*'s local consumers will wish to make a non-local search and so firm *i*'s local fresh demand becomes equal to (1/n) and its local return demand must equal zero. If $p_{i}>p_{T}$, any non-local consumer who visits firm *i* will never wish to stop, such that firm *i*'s non-local fresh demand becomes equal to zero and its non-local return demand must be re-expressed. Further kinks will also be present at the more extreme prices, pB^=p⁎+ε̲−x^ and pT^=p⁎+ε¯−(x^−s). Consequently, a general proof of existence appears intractable in this model but existence can be demonstrated within the special case where s→0. Here, the model collapses to some standard search models and so we can apply previous results. When $p^{⁎}<\underset{̲}{\varepsilon }$ existence is demonstrated by Anderson and Renault (1999). When $p^{⁎}>\underset{̲}{\varepsilon }$, although Wolinsky (1986) did not consider such kinks, Armstrong et al. (2009) prove existence in the special case of a standard uniform match distribution.

## Appendix C Proofs of Propositions

Proof of Proposition 1 When a symmetric equilibrium exists, the equilibrium price must form a solution to the necessary first order condition, $p^{⁎}=-D_{i}(p^{⁎},p^{⁎})/D_{i}^{\prime }(p^{⁎},p^{⁎})$. To see that the price in Proposition 1 follows directly from this condition, first note ∑k=0n−2G(x^)k=(1−G(x^)n−1)/(1−G(x^)). Using this and (3)–(6), then note that when evaluated at $p_{i}=p^{⁎}$, (i) $D_{i}(p_{i},p^{⁎})$ in (2), implies $D_{i}(p^{⁎},p^{⁎})=(1/n)[1-G(\max\{\underset{̲}{\varepsilon },p^{⁎}\})G{\left(\max\{\underset{̲}{\varepsilon },p^{⁎}\}+s\right)}^{n-1}]$ and (ii) $D_{i}^{\prime }(p_{i},p^{⁎})$ implies Di′(p⁎,p⁎)=−(1/(n(ε¯−ε̲))·[1+G(x^−s)∑k=0n−2G(x^)k−I(p⁎≤ε̲)G(ε̲+s)n−1]. Further, for any symmetric equilibrium to exist, a solution to the first order condition must lie within p⁎∈[0,x^−s) as Condition 1 implies p⁎<x^−s. Conditional on the existence of such a solution, the equilibrium price is unique. This is most easily observed if one rearranges the FOC as $\left(p^{⁎}/D_{i}(p^{⁎},p^{⁎}))=-D_{i}^{\prime }(p^{⁎},p^{⁎}\right)$ and notes that the left-hand side is independent of x^ while the right-side is strictly increasing in x^ for all x^−s∈(ε̲,ε¯), which is ensured via Condition 1 so long as either c>0 or s>0.

Proof of Proposition 2 We will first show that $\partial p^{⁎}/\partial c>0$ for all n≥2. Using (9) and denoting $p_{\mathrm{NC}}^{⁎}$ as $p^{⁎}$ for convenience, define H=(p⁎/(ε¯−ε̲))[1+G(x^−s)∑k=0n−2G(x^)k)]−[1−G(p⁎)G(p⁎+s)(n−1)]=0. From the implicit function theorem, $\partial p^{⁎}/\partial c=-(\partial H/\partial c)/(\partial H/\partial p)$. Under our assumptions with non-market coverage, ε̲<p⁎<x^−s≤ε¯, one can show that ∂H/∂p>0. Therefore the sign of $\partial p^{⁎}/\partial c$ depends on the sign of −∂H/∂c=−(p⁎/(ε¯−ε̲))[G(x^−s)·(∂∑k=0n−2G(x^)k/∂c)+∑k=0n−2G(x^)k·(∂G(x^−s)/∂c)], or equivalently, −(p⁎/(ε¯−ε̲)2)[G(x^−s)∑k=0n−2kG(x^)k−1+∑k=0n−2G(x^)k](∂x^/∂c). This is positive for all n≥2 as ∂x^/∂c=−(ε¯−ε̲)/(ε¯−x^))<0. Finally, we will now show that $\partial p_{\mathrm{NC}}^{⁎}/\partial s>0$ for all n>2, and for n=2 if $\underset{̲}{\varepsilon }>0$. From above, we know that the sign of $\partial p^{⁎}/\partial s$ depends on the sign of −∂H/∂s=−(p⁎/(ε¯−ε̲))[∑k=0n−2G(x^)k·(∂G(x^−s)/∂s)]+[∂(1−G(p⁎)G(p⁎+s)n−1)/∂s] or equivalently, (ε¯−ε̲)−1[(p⁎/(ε¯−ε̲))∑k=0n−2G(x^)k−G(p⁎)(n−1)G(p⁎+s)n−2]. We will now show that this is zero when n=2 and $\underset{̲}{\varepsilon }=0$, but positive for all other valid parameters. First, compare the terms $\left(p^{⁎}/(\overset{¯}{\varepsilon }-\underset{̲}{\varepsilon })\right)$ and $G(p^{⁎})$. As $G(p^{⁎})$ can be written as $\left(p^{⁎}-\underset{̲}{\varepsilon })/(\overset{¯}{\varepsilon }-\underset{̲}{\varepsilon }\right)$, note that $\left(p^{⁎}/(\overset{¯}{\varepsilon }-\underset{̲}{\varepsilon }))=G(p^{⁎}\right)$ when $\underset{̲}{\varepsilon }=0$ and $\left(p^{⁎}/(\overset{¯}{\varepsilon }-\underset{̲}{\varepsilon }))>G(p^{⁎}\right)$ when $\underset{̲}{\varepsilon }>0$. Now compare the remaining terms ∑k=0n−2G(x^)k and $(n-1)G{\left(p^{⁎}+s\right)}^{\left(n-2\right)}$, and note that ∑k=0n−2G(x^)k=(n−1)G(p⁎+s)(n−2) when n=2 and ∑k=0n−2G(x^)k>(n−1)G(p⁎+s)(n−2) when n>2 because G(x^)>G(p⁎+s).

Proof of Proposition 3 Denoting $p_{\mathrm{NC}}^{⁎}$ as $p^{⁎}$, we want to demonstrate that $(\partial p^{⁎}/\partial c)-(\partial p^{⁎}/\partial s)>0$. Using the logic and results from Proposition 2, this requires (−∂H/∂c)−(−∂H/∂s)>0. With some rearrangement of the expressions above, this condition holds if −(p⁎/(ε¯−ε̲))[G(x^−s)·(∂∑k=0n−2G(x^)k/∂c)]−(p⁎/(ε¯−ε̲))[∑k=0n−2G(x^)k·((∂G(x^−s)/∂c)−(∂G(x^−s)/∂s))]−(∂(1−G(p⁎)G(p⁎+s)n−1)/∂s)>0, or equivalently, if −(p⁎/(ε¯−ε̲))[G(x^−s)∑k=0n−2kG(x^)k−1·(∂x^/∂c)]−(p⁎/(ε¯−ε̲))[∑k=0n−2G(x^)k·((∂x^/∂c)+1)]+(n−1)G(p⁎)G(p⁎+s)(n−2)>0. This is true for all *n* as (∂x^/∂c)=−(ε¯−ε̲)/(ε¯−x^))<−1.

Proof of Proposition 4 To show that the price under market coverage, (8), is increasing in *c*, note that ∂(1/pC⁎)/∂c=(ε¯−ε̲)−2(∂x^/∂c)[∑k=0n−2G(x^)k+G(x^−s)∑k=0n−2kG(x^)k−1]. Under the assumptions with market coverage, pC⁎≤ε̲<x^−s≤ε¯, this is negative for all n≥2 because ∂x^/∂c=−(ε¯−ε̲)/(ε¯−x^))<0.To show that the price is also increasing in *s*, note that ∂(1/pC⁎)/∂s=−(ε¯−ε̲)−2[∑k=0n−2G(x^)k+(n−1)G(ε̲+s)n−2] is negative for all n≥2.

Proof of Proposition 5 Define $A=\partial (1/p_{C}^{⁎})/\partial c-\partial (1/p_{C}^{⁎})/\partial s$. Search (switching) costs will have the larger relative marginal effect if *A* is negative (positive). By rearranging the expressions from the proof of Proposition 4, *A* can be presented as follows.

A·(ε¯−ε̲)2=G(x^−s)∑k=0n−2kG(x^)k−1·∂x^∂c+∑k=0n−2G(x^)k·∂x^∂c+1+(n−1)G(ε̲+s)n−2

By using a series of steps, we will now show that A<0 for all ‘valid’ parameters whenever n≥4. Valid parameters are defined as those that are consistent with the model's assumptions under market coverage, such that pC⁎≤ε̲<x^−s≤ε¯.
Step 1. As (∂x^/∂c)<0, it is trivial to show that ∂A/∂s>0. Further, we know that in any valid set of parameters, s<x^−ε̲. Therefore, *A* will always be less than As=x^−ε̲ for all valid values of *s*, where As=x^−ε̲·(ε¯−ε̲)2=∑k=0n−2G(x^)k·((∂x^/∂c)+1)+(n−1)G(x^)n−2. Therefore, to demonstrate that A<0 for all n≥4, we now only need to show that As=x^−ε̲≤0 for all n≥4. We proceed to show that this is true for two exhaustive cases (i) when x^>(ε¯+ε̲)/2 in Step 2 and (ii) x^≤(ε¯+ε̲)/2 in Step 3.
Step 2. Let x^>(ε¯+ε̲)/2. It then follows that ((∂x^/∂c)+1)=−(x^−ε̲)/(ε¯−x^)<−1. As ∑k=0n−2G(x^)k≥(n−1)G(x^)n−2, we then know that As=x^−ε̲≤0 for all *n*.
Step 3. Now let x^≤(ε¯+ε̲)/2.
Step 3a. It can be shown that As=x^−ε̲ is decreasing in *n* if G(x^)<e−(1/(n−1)). To see this, note that the first term of As=x^−ε̲ is clearly decreasing in *n* as ((∂x^/∂c)+1)<0 and that the second term is also decreasing in *n* when ∂(n−1)G(x^)n−2/∂n=G(x^)n−2[1+(n−1)lnG(x^)]<0.
Step 3b. It then follows that As=x^−ε̲ is decreasing in *n* for all n≥4. To see this, note that the condition G(x^)<e−(1/(n−1)) becomes easier to satisfy as *n* increases. Thus, if it holds for n=4 it will hold for all n≥4. When n=4, it is indeed satisfied because we know that G(x^)≤0.5 for all x^≤(ε¯+ε̲)/2 and that $e^{-(1/3)}>0.71$.
Step 3c. Finally, as we now know that As=x^−ε̲ is decreasing in *n* for all n≥4, to ensure that *A* remains negative for all n≥4 we need only show that As=x^−ε̲≤0 when n=4. With simplification, we therefore require As=x^−ε̲,n=4·(ε¯−ε̲)2=[1+G(x^)+G(x^)2][(∂x^/∂c)+1]+3G(x^)2≤0 for all x^∈(ε̲,(ε¯+ε̲)/2]. This can be shown to be true most easily by rewriting (∂x^/∂c)+1=−(x^−ε̲)/(ε¯−x^) as −G(x^)/(1−G(x^)), such that As=x^−ε̲,n=4·(ε¯−ε̲)2=−[G(x^)/(1−G(x^))][4G(x^)2−2G(x^)+1] which is negative for all G(x^)∈(0,0.5].

Proof of Proposition 6 Following from the explanation of Proposition 6 in the text, it only remains to show that the aggregate number of non-local searches, $K_{c}$, is larger than the total number of switches, $K_{s}$, in equilibrium. If n<∞, we know this is true because (i) switching consumers switch only once, (ii) all switchers must conduct at least one non-local search and (iii) a positive proportion of consumers search beyond their local firm yet decide not to switch. This latter proportion equals the market level of local return demand, ${\mathrm{nR}}_{\mathrm{Li}}(p^{⁎},p^{⁎})$, which from (5), equals (ε¯−ε̲)−1∫max{ε̲,p⁎}+sx^G(ε)n−1dε>0. If n=∞, all return demand tends to zero as all searching consumers eventually find an attractive offer and switch. Hence, the proportion of consumers who search then equals the proportion of consumers who switch. However, we know that, on average, each searching consumer conducts more than one search. Indeed, the average number of searches made by a searching consumer equals (1−G(x^))[1+2G(x^)+3G(x^)2…+(n−1)G(x^)n−2]+(n−1)G(x^)n−1 or equivalently, ∑k=0n−2G(x^)k, which is greater than one when n=∞ if x^>ε̲, as ensured via Condition 1.

Proof of Proposition 7 To avoid a monopoly price equilibrium, we must assume that some non-local search takes place which now requires max{0,ε̲−p1⁎}<x^−s−p2⁎. Given n→∞, there is no return demand. Therefore, as all consumers are local to Firm 1, its demand derives solely from the consumers that are unwilling to start a non-local search. Given a price at Firm 1, $p_{1}$, and a correct expectation of a non-local price, $p_{2}^{⁎}$, it follows that D1(p1,p2⁎)=1−G(x^−s+p1−p2⁎). Now consider the (n−1) entrants who have no local consumers. Their demand can only result from the G(x^−s+p1⁎−p2⁎) consumers who start a search from Firm 1. Such consumers will visit any given entrant firm, say Firm 2, with probability, (1/(n−1))∑k=0n−2G(x^)k, and find it optimal to stop at Firm 2 with probability, 1−G(x^+p2−p2⁎), such that D2(p2;p1⁎,p2⁎)=G(x^−s+p1⁎−p2⁎)·[1/(n−1)]·[∑k=0n−2G(x^)k]·[1−G(x^+p2−p2⁎)]. After stating the incumbent and entrant profit maximisation problems and simultaneously solving the two first order conditions, it follows that p1⁎=ε¯−x^+(s/2) and p2⁎=ε¯−x^. From the assumption that some consumers search in equilibrium, max{0,ε̲−p1⁎}<x^−s−p2⁎, we know that x^>ε̲+(s/2). This, together with c>0, then ensures that $\partial p_{i}^{⁎}/\partial c>\partial p_{i}^{⁎}/\partial s$ for i=1,2. By noting that industry profits, Π, equal $p_{1}^{⁎}D_{1}(p_{1}^{⁎},p_{2}^{⁎})+p_{2}^{⁎}[1-D_{1}(p_{1}^{⁎},p_{2}^{⁎})]$, one can then further show ∂Π/∂c>∂Π/∂s. Finally, using similar arguments to that used in Proposition 6, one can demonstrate that the results for total welfare and consumer surplus follow for c>0 if the total number of searches, $K_{c}$, exceeds the total number of switches, $K_{s}$. Here, this is ensured as the average switcher conducts [1/(1−G(x^))]>1 searches.

Proof of Proposition 8 To avoid a monopoly price equilibrium, we must assume that some non-local search takes place which now requires max{0,ε̲−pL⁎}<x^−s−pNL⁎. Given n→∞, there is no return demand. Consider any firm *i*. Its $a_{i}$ local consumers can buy at its local price, $p_{\mathrm{iL}}$. However, such consumers will only buy if they do not initiate a non-local search. Given a correct expectation of the non-local price, $p_{\mathrm{NL}}^{⁎}$, firm *i*'s local demand will then equal DiL(piL,pNL⁎)=ai[1−G(x^−s+piL−pNL⁎)]. Now consider firm *i*'s non-local demand. Any of the $\left(1-a_{i}\right)$ consumers that are not local to Firm *i* can buy from firm *i* at the price, $p_{\mathrm{iNL}}$. Such consumers will start to search beyond their own local firm with probability, G(x^−s+pL⁎−pNL⁎), visit firm *i* with probability, ∑k=0n−2G(x^)k, and find it optimal to stop at firm *i* with probability, 1−G(x^+piNL−pNL⁎), such that DiNL(piNL;pNL⁎,pL⁎)=(1−ai)·G(x^−s+pL⁎−pNL⁎)·∑k=0n−2G(x^)k·[1−G(x^+piNL−pNL⁎)]. Firm *i*'s profits are then equal to $p_{\mathrm{iL}}D_{\mathrm{iL}}(.)+p_{\mathrm{iNL}}D_{\mathrm{iNL}}(.)$. After stating the associated maximisation problem and simultaneously solving the two first order conditions, it follows that pL⁎=ε¯−x^+(s/2) and pNL⁎=ε¯−x^. From the assumption that some consumers search in equilibrium, max{0,ε̲−pL⁎}<x^−s−pNL⁎, we know that x^>ε̲+(s/2). This, together with c>0, then ensures that $\partial p_{L}^{⁎}/\partial c>\partial p_{L}^{⁎}/\partial s$ and $\partial p_{\mathrm{NL}}^{⁎}/\partial c>\partial p_{\mathrm{NL}}^{⁎}/\partial s$. By noting that industry profits, Π, equal $p_{L}^{⁎}\cdot \sum_{i=1}^{n}a_{i}D_{\mathrm{iL}}(p_{L}^{⁎},p_{\mathrm{NL}}^{⁎})+p_{\mathrm{NL}}^{⁎}\cdot (1-\sum_{i=1}^{n}a_{i}D_{\mathrm{iL}}(p_{L}^{⁎},p_{\mathrm{NL}}^{⁎}))$, one can further show ∂Π/∂c>∂Π/∂s. Finally, using similar arguments to that used in Proposition 6, one can show that the results for total welfare and consumer surplus follow for c>0 if the total number of searches, $K_{c}$, exceeds the total number of switches, $K_{s}$. Here, this is ensured as the average switcher conducts [1/(1−G(x^))]>1 searches.

## Footnotes

1 For more see http://nobelprize.org/nobel_prizes/economics/laureates/.

2 Waterson (2003) offers a useful overview. For more detailed reviews see Baye et al. (2006) on search costs and Klemperer (1995) or Farrell and Klemperer (2007) on switching costs.

3 Related issues have also occurred in the UK mobile phone market where the regulator has recently introduced legislation to improve the switching process by requiring providers to port switching consumers' phone numbers to their new provider within one working day (in line with similar moves by the EU and US), while at the same time expressing concerns over the accessibility of price information (OFCOM, 2010). Other UK cases where both search and switching costs have been an important issue include the investigations into extended warranties, storecards and payment protection insurance.

4 The use of a framework with product differentiation prompts the existence of a pure-strategy price equilibrium and contrasts to the more traditional search models of price dispersion (e.g. Stahl, 1989). Although similar results can also be produced in a price dispersion model, the chosen framework is more suited to examining the effects of the two costs by providing a fuller analysis of consumers' search behaviour. Related frameworks are being used increasingly to study other issues including prominence (Armstrong et al., 2009) and advertising (Haan and Moraga-González, 2011).

5 Using our latter terminology, these features limit the comparison of effects by minimising the impact of Distinctions 2, 3 and 4.

6 These date back to Calem and Mester (1995) who offered support for Ausubel's (1991) assertion that price stickiness and supranormal profits in the credit card market could be explained by the existence of search costs and switching costs. Further contributions include Knittel (1997) who demonstrates a positive relationship between price-cost margins and proxies for search costs and switching costs in the US telephone market, and Giulietti et al. (2005) who show how proxies are negatively related to the propensity for consumers to switch in the UK gas market. In a different vein, Giulietti et al. (2010) use observations of tariff dispersion within the UK electricity market to make some separate inferences about the trends of search and switching costs over time.

7 While not providing simultaneous estimates, there are two other related papers. Dubé et al. (2010) provide a clever money-metric estimate of loyalty which they interpret as a switching cost rather than a search cost because it remains unaffected by the presence of in-store advertising, while Moshkin and Shachar (2002) estimate that 71% of individuals' television viewing patterns are more consistent with the existence of search costs rather than switching costs.

8 Asymmetric configurations and the impact of positive local search costs are considered in Section 7.

9 Dynamic effects and the possibility of price discrimination are discussed separately in Section 7.

10 Due to the assumption that c≥0, it must be that x^≤ε¯. Further, if x^<ε̲ then search cannot be optimal and x^ can be set equal to $\underset{̲}{\varepsilon }$ without loss.

11 To show why the presented stopping rule is optimal more generally for β≥1, first suppose β=2. If ε<x^, it must remain optimal to search - if search is optimal when β=1 then it must also be optimal when β=2. If ε≥x^, the presented stopping rule suggests stopping. If instead, the consumer chose to search, it would be optimal to search only once. To understand why, note that after making one search, only one unsearched firm would remain (β=1) and, as the consumer would have a best match value of at least x^, it would be optimal to stop. Hence, the decision of whether to further search when β=2 is, in fact, only a decision between stopping immediately and making one more search, where the presented stopping rule has already been shown to be optimal. This argument can then be expanded for higher levels of β.

12 To motivate his empirical analysis, Knittel (1997) also provides a theoretical description of consumers' optimal behaviour under search and switching costs. However, his analysis only goes as far as presenting the equivalent of our Step 1.

13 In parallel to footnote 10, the assumptions that c,s≥0 ensure x^−s≤ε¯. If x^−s<ε̲ then search cannot be optimal and x^ can be set equal to $\underset{̲}{\varepsilon }+s$ without loss.

14 Larger price deviations can alter the form of residual demand and prevent the profit function from being globally concave. This is later discussed within the context of equilibrium existence in Appendix B.

15 That is, apart from an odd special case when n=2 and $\underset{̲}{\varepsilon }=0$ where an increase in switching costs has no effect on the price, as further explained below.

16 From Proposition 2, we know that this price-decreasing effect of switching costs is dominated by its price-increasing effect on deterring non-local search in all but one case. There, when n=2 and $\underset{̲}{\varepsilon }=0$, the two effects exactly offset each other such that an increase in switching costs has no effect on the price.

17 In particular, consider a market coverage equilibrium. From previous results we know a consumer will be indifferent over starting a non-local search when εi−p⁎=x^−s−p⁎. Hence, the expected benefit from searching can be considered equal to x^−s−p⁎. The expected benefit from switching to a random alternative without searching equals $E(\varepsilon )-s-p^{⁎}$. Searching will be preferred when x^>E(ε) or $c<(\overset{¯}{\varepsilon }-\underset{̲}{\varepsilon })/8$.

18 Alternative equilibrium conditions can be used. However, most alternatives have the drawback of producing measures that require more data than those derived below, being dependent upon the number of firms, the market coverage assumption and potentially, the equilibrium price.

19 Honka (2010) makes excellent progress in this direction. By identifying search costs with the use of more detailed data on the extensiveness of individual's search activity, she is able to report no bias in the search-cost-only version of her methodology to estimate search costs and switching costs simultaneously.

20 The details and findings of the survey are provided in Chang and Waddams Price (2008).

21 The choice of a relevant decision period is also an issue within Kim et al. (2003) where switching costs are estimated using data on switching rates, prices and costs within a market for bank loans. They use a three year period and provide additional market evidence to support this choice.

22 A very crude comparison of the magnitude of the two costs can be made by assessing the findings of single-cost studies in different markets. For example, switching costs are estimated by Shy (2002) to be equal to $400–464 ($227–313) within the Finish bank market (Israeli cellular phone market), and equal to $109–186 within the US television market by Shcherbakov (2009), while De los Santos et al. (forthcoming) estimate search costs of $1.35 in the US online book market and Moraga-González and Wildenbeest (2008) propose search costs of $2.21–12.26 for 70% of consumers in the online market for memory chips. A much better comparison is provided by Honka's (2010) unique study of both costs in the US auto insurance industry. She finds switching costs of $85 and search costs of $45–110. However, her data is qualitatively different to ours with almost all consumers choosing to search.

23 In terms of the theoretical equilibrium more generally, one can check that the introduction of γ raises the equilibrium price but leaves the main comparative statics unchanged.
